# Polysaccharide/Silica Microcapsules Prepared via Ionic Gelation Combined with Spray Drying: Application in the Release of Hydrophilic Substances and Catalysis

**DOI:** 10.3390/polym15204116

**Published:** 2023-10-17

**Authors:** Asmaa M. Elzayat, Inés Adam-Cervera, Marie Albus, Amparo Cháfer, José D. Badia, Francisco F. Pérez-Pla, Rafael Muñoz-Espí

**Affiliations:** 1 Institute of Materials Science (ICMUV), Universitat de València, C/Catedràtic José Beltrán 2, 46980 Paterna, Spain; 2 Physics Department, Faculty of Science, Mansoura University, Mansoura 35516, Egypt; 3 Department of Chemical Engineering, School of Engineering, Universitat de València, Av. de la Universitat s/n, 46100 Burjassot, Spain

**Keywords:** chitosan, alginate, ionic gelation, silica, microparticle, spray drying, drug release, heterogeneous catalysis

## Abstract

Polysaccharide/silica hybrid microcapsules were prepared using ionic gelation followed by spray-drying. Chitosan and alginate were used as biopolymer matrices, and in situ prepared silica was used as a structuring additive. The prepared microparticles were used in two very different applications: the encapsulation of hydrophilic molecules, and as a support for palladium nanoparticles used as catalysts for a model organic reaction, namely the reduction of *p*-nitrophenol by sodium borhydride. In the first application, erioglaucine disodium salt, taken as a model hydrophilic substance, was encapsulated in situ during the preparation of the microparticles. The results indicate that the presence of silica nanostructures, integrated within the polymer matrix, affect the morphology and the stability of the particles, retarding the release of the encapsulated substance. In the second application, chloropalladate was complexed on the surface of chitosan microparticles, and palladium(II) was subsequently reduced to palladium(0) to obtain heterogeneous catalysts with an excellent performance.

## 1. Introduction

Biopolymer-based microparticles have gained significant attention as drug delivery systems as a result of their wide range of properties and applications. The use of microparticle-based therapy allows drug release to be carefully targeted to the specific treatment site through the choice and formulation of various drug–polymer combinations. By using microencapsulation technologies and varying the formulation parameters, polymeric microspheres can be developed into an optimal drug delivery system, which should provide the desired release profile [1,2,3,4].

Spray-drying has been widely used in recent times in the pharmaceutical and food industries for the preparation of microparticles; because it is a continuous process, it can be easily scaled up, and it has a relatively low processing cost [5]. Through the transformation of a liquid feed into dry powders, spray-drying allows the control of particle size by tuning the processing parameters [6,7]. The process starts with the atomization of the fluid feed inside a chamber at a high temperature, through a peristaltic pump to an atomizer or nozzle [8]. Afterward, atomization is performed via centrifugation, pressure or kinetic energy due to the force of the compressed air, which disrupts the liquid and splits it into small droplets. The transformation of fine droplets to dry particles takes place with the help of both a heated gas stream and fast solvent evaporation, resulting in the separation and collection of the dried particles in a glass cyclone collector [9,10,11,12].

Conventional polysaccharide microspheres prepared via spray-drying cannot be kept suspended in water because of their great swelling and dissolution. Accordingly, non-cross-linked microspheres are unsuitable for sustained delivery systems. Chitosan and alginate are two common non-toxic and biocompatible hydrophilic polysaccharides that can easily swell in aqueous media. They can form gels by interacting with different types of polyvalent anions (in the case of chitosan) or cations (in the case of alginate). Ionic gelation has also been explored to overcome certain disadvantages of chemical cross-linkers, such as toxicity [13,14]. Cross-linking agents are therefore required to prepare stabilized microparticles [15,16,17]. In the case of chitosan, triphosphate is a nontoxic polyanion that interacts in acidic media via electrostatic forces to form ionically cross-linked networks. In case of alginate, CaCl_2_ has shown outstanding performance as a physical cross-linker [18].

The incorporation of silica has been recognized as safe by the US Food and Drug Administration (FDA). Silica has emerged as a component for promising drug carriers because it is nearly nontoxic, biocompatible, and easily surface-modified [19]. Silica nanoparticles in suspension can be formed by sol−gel processes in the presence of polysaccharides, yielding the formation of a hybrid polymer structure with potential uses in the delivery of therapeutic agents [20,21,22,23]. Organic–inorganic hybrid structures are able to protect the encapsulated payloads from the surrounding environment, and allow their release in a controlled manner [24,25,26,27].

Besides their mentioned uses in drug release and biomedicine, polysaccharides have also been shown to have a great potential in a completely different field: heterogeneous catalysis [28,29,30]. Different polysaccharide supports have been used to load noble metals for catalysis [31]. In particular, palladium is an important noble metal catalyst used in different organic reactions. It has been demonstrated that the material and structure of the support play a significant role in the catalytic performance. In addition, the magnitude of the interaction between the support and the Pd^2+^ ions has a great influence on the catalytic activity of the resulting metal nanoparticles [32,33]. Chitosan offers a strong ability to chelate metal ions [34]. In this respect, chitosan supports have shown good interaction with palladium through coordination by heteroatoms, such as N and O, on the branches of the polymer molecules [35,36].

In this work, we report the preparation of polysaccharide microparticles based on chitosan and alginate, containing silica nanostructures embedded within their matrix, via ionic gelation followed by a spray-drying process. Various formulations were prepared and tested in terms of the morphology and structural stability of the resulting particles. Chitosan microparticles were selected for studying two different applications. First, erioglaucine disodium salt was encapsulated in situ during the preparation of the microparticles, and their release behavior was studied in a neutral medium. Second, the microparticles were also applied as a catalyst support for the reduction of *p*-nitrophenol by NaBH_4_. This reaction has been mostly applied because of their low energy requirements, economy, effectiveness, safety, and ease of operation. Chloropalladate was complexed on the surface of chitosan microparticles, and palladium(II) was subsequently reduced to palladium(0) to obtain heterogeneous catalysts with an excellent activity. The results indicate that microparticles prepared using this process exhibited great performance in both applications, long-term stability, and good mechanical strength.

## 2. Materials and Methods

### 2.1. Materials

All of the following chemicals and solvents were used as received: low-molecular-weight chitosan (Sigma-Aldrich/Merck, Darmstadt, Germany, *M*_v_ = 50,000–190,000 Da, 75–85% deacetylated); alginic acid sodium salt (powder, 15–25 cP, 1% in H_2_O); glacial acetic acid (Panreac-Química, Barcelona, Spain); sodium triphosphate (STP, Alfa-Aesar, Karlsruhe, Germany, ≥85%); calcium chloride dihydrate (Sigma-Aldrich/Merck, extra pure); tetraethyl orthosilicate (TEOS, Sigma-Aldrich/Merck, ≥98%); hydrochloric acid 37% (J. T. Baker, Phillipsburg, USA); ethanol (Merck, Darmstadt, Germany, gradient grade for liquid chromatography); erioglaucine disodium salt (Across Organics, Geel, Belgium, pure); phosphate-buffered solution (pH = 7.4) self-prepared with NaCl (Sigma-Aldrich/Merck, ≥99%); potassium chloride (Sigma-Aldrich/Merck, ≥99%); disodium hydrogenphosphate monohydrate (Schlarlau, Hamburg, Germany, reagent grade) and potassium dihydrogenphosphate (Sigma-Aldrich/Merck, ≥99%); palladium(II) chloride (Sigma-Aldrich/Merck, 99%); sodium borohydride (Sigma-Aldrich/Merck, 99%); and *p*-nitrophenol (Doesder, Barcelona, Spain). Ultrapure water was used through all the experiments.

### 2.2. Formation of Polysaccharide/Silica Hybrid Microcapsules

Chitosan and alginate microcapsules were prepared using a spray-drying process combined with ionotropic gelation, through which an electrostatic interaction between polyelectrolyte and an oppositely charged cross-linking agent takes place.

Chitosan is a cationic polyelectrolyte with amino groups (–NH_3_^+^) that can interact with the anions of the cross-linker (P_3_O_10_^5−^). Alginate, in contrast, possesses carboxylate groups (COO^−^) that are able to interact with Ca^2+^ ion. To prepare chitosan/silica and alginate/silica hybrid microcapsules, tetraethyl orthosilicate (TEOS) was added to a polysaccharide solution, yielding silica nanostructures upon contact with water through a sol–gel process. The forming silica is trapped in the biopolymer matrix, leading to hybrid polymer/silica structures. Briefly, a 0.3 wt% chitosan solution was prepared by dissolving the polymer in a 2% *v*/*v* acetic acid aqueous solution. The solution was stirred for 24 h at room temperature. In the case of the samples with silica (and only for those samples), 100 μL of concentrated hydrochloric acid (37%) was added to 200 mL of the previously prepared chitosan solution and stirred for 1 h. Then, TEOS was added at a rate of 3:4 (wt/wt) in relation to chitosan. The resulting solution was stirred for 4 h at room temperature. The cross-linker solution was prepared in deionized water at a concentration of 3 wt%. Chitosan colloids were spontaneously fabricated after dropwise addition of the cross-linker to the chitosan solution at a ratio of chitosan to sodium triphosphate (STP) of 2:1 (wt/wt) under magnetic stirring for 8 h at room temperature. This chitosan-to-STP ratio was selected based on our previous studies [27,37]; these conditions provided an opalescent suspension, which is indicative of optimum cross-linking. The chitosan or the chitosan/silica hybrid microparticles were hardened and spray-dried using a Büchi Mini Spray Dryer B-191 (Flawil, Switzerland). The operating parameters were set as follows: the inlet air temperature was in the range of 110 °C, the liquid flow rate was between 150 and 200 mL/h, and the aspiration rate was about 80%. The equipment operated in co-current flow mode. To maintain homogeneity, the colloidal solution was kept under magnetic stirring during the spray-drying process.

The solid capsules were collected from the apparatus collector and stored under a vacuum at room temperature. The same strategy was carried out during the encapsulation of a hydrophilic molecule, namely erioglaucine disodium salt, within the polymer/silica network structure.

Alginate microcapsules were analogously prepared following the same procedure, but using CaCl_2_ as a physical cross-linker. For samples containing silica (and not for samples with alginate alone), 200 µL of concentrated NH_4_OH was added to the alginate aqueous solution before the addition of TEOS.

### 2.3. Encapsulation of Erioglaucine Disodium Salt

Erioglaucine disodium salt was employed as a payload molecule to study the release from chitosan-based microcapsules in phosphate buffer (pH = 7.4) at 37 °C. The hydrophilic molecule (2 wt%) was added during the initial step to a chitosan aqueous solution either in the presence or in the absence of silica with constant stirring at 800 rpm for 2 h. The cross-linker was added dropwise to the chitosan solution at a chitosan-to-STP ratio of 2:1 (wt/wt), under magnetic stirring for 8 h at room temperature. When the liquid was fed to the nozzle with a peristaltic pump, atomization occurred by the force of the compressed air, disrupting the liquid into small droplets. The droplets, together with hot air, were blown into a chamber wherein the solvent in the droplets was evaporated and discharged out through an exhaust tube. The dry product was then collected in a collection glass path.

### 2.4. Particle Characterization

#### 2.4.1. Water Uptake and Swelling Capacity

A known weight of spray-dried cross-linked microspheres (100 mg) was placed in a phosphate-buffered solution (pH = 7.4) for a period of 8 h. The swollen microspheres were collected via centrifugation, and the wet weight of the swollen microspheres was determined by blotting the particles with filter paper to remove water absorbed on the surface and weighing them immediately after that. The weight of the swollen microspheres was recorded at a pre-determined time period (2, 4, 6, and 8 h). The percentage swelling capacity of the micro-spheres in the dissolution media was then calculated using the following expression:

(1) $$Masss\;welling\;(\%)=\frac{w_{w}}{w_{d}}\times 100$$

where *w*_w_ is the wet weight of particles at a certain time, and *w*_d_ is the initial weight of the dried particles. Data points are the average of three measurements.

#### 2.4.2. Morphology, Composition, and Surface Area

The surface morphology of the microcapsules was investigated via scanning electron microscopy (SEM) using a Hitachi S4800 microscope (Tokyo, Japan). The powders were previously fixed on a brass stub using double-sided adhesive tape, and then were made electrically conductive by coating under a vacuum with a thin layer of platinum (approximately 3–5 nm) for 3 min at 30 W. Images were taken in decelerating mode at a voltage of 0.5 kV. Elemental mapping was carried out via energy dispersive X-ray analysis (EDX) to provide the spatial distribution and composition of the palladium in the samples.

Nitrogen adsorption–desorption isotherms were recorded using an automated Micromeritics ASAP2020 instrument (Norcross, GA, USA). Prior to the adsorption measurements, the samples were outgassed in situ under a vacuum (10–6 Torr) at 120 °C for 15 h to remove adsorbed gases.

#### 2.4.3. Thermogravimetric Analysis

Thermogravimetric analysis (TGA) was carried out with a TGA-50 thermobalance (TA Instruments, New Castle, DE, USA) by heating the samples from room temperature to 1000 °C at a heating rate of 10 °C min^−1^ under air atmosphere.

### 2.5. Release Study

The release of the entrapped hydrophilic molecules (erioglaucine) was assessed using a dialysis membrane method. The loaded chitosan microparticles were placed in a dialysis membrane (MWCO 14 kDa, Carl Roth, Karlsruhe, Germany) containing 5 mL of an aqueous phosphate buffer (pH = 7.4), subsequently sealed, and placed in thermo-jacketed glass vessel containing 200 mL of the same buffer at a controlled temperature of 37 °C. The receptor solution was homogenized by means of magnetic stirring at 300 rpm. The volume enclosed within the dialysis membrane is significantly smaller than the outer volume. Dye molecules liberated from the microcapsules diffuse through the dialysis membrane to the outer compartment. At defined time intervals, aliquots were extracted and replaced by the same fresh volume. The samples were then assayed spectrophotometrically by measuring the maximum of absorbance at 629 nm (Jasco V-777 spectrophotometer, Tokyo, Japan). The concentration of the dye was determined using a calibration curve. The release profiles were fitted and evaluated according to an empiric model known as the Hill equation, as used in previous work [27,37],

(2) $$\hat{Q}\left(t\right)=\frac{Q_{max}t^{n}}{t_{\frac{1}{2}}+t^{n}}$$

where *Q*_max_ is the maximum percentage of released load at time infinity, *t*_1/2_ is the time required for 50% of the load to be released, and *n* is a sigmoidicity exponent. Fixing the value of *n* to 1 implies a simple sigmoidal model analogous to a Michaelis–Menten-type kinetics. The Hill equation (and its simplified Michaelis–Menten form) is formally analogous to the Langmuir model in heterogeneous catalysis, and it has been previously used to analyze quantitative drug–receptor relationships [38]. This model can be used as a convenient empiric relationship to fit release data as a function of time [39].

### 2.6. Deposition of Pd(0) Nanoparticles on of Chitosan Microparticles

The prepared chitosan microparticles were functionalized with Pd(0) through a post-loading method. First, chitosan and chitosan/silica microparticles were loaded with Pd^2+^ ions. For this purpose, PdCl_2_ (0.004 M) was dissolved in an aqueous solution of hydrochloric acid (0.1 M, pH = 1), and a specific amount of microparticles (50 mg) were added to this solution. The resulting suspension was stirred for 3 h at 800 rpm, and the pH was raised to 12 by adding NaOH. The mixture was further stirred at room temperature for 12 h. Afterwards, the particles were separated via centrifugation (10,000 rpm, 10 min) and placed in an aqueous solution of NaBH_4_ (0.075 M), which was stirred for 4 h until the reduction of Pd(II) was completed. The particles were then separated again via centrifugation, washed with distilled water, and dried at 50 °C under reduced pressure.

### 2.7. Catalytic Experiments

All catalysis experiments were carried out under a protective argon atmosphere with overpressure. Before the experiments, an aqueous stock solution of *p*-nitrophenol (0.001 M) was flushed with argon for 30 min at 25 °C in an ultrasound bath to expel any dissolved oxygen. A volume of this *p*-nitrophenol solution (5 mL, 0.005 mmol) was added to a vial containing the catalytic particles (~5 mg) and NaBH_4_ (1.5 mmol, final concentration of 0.3 M). The reaction mixture was then stirred at 700 rpm at 25 °C in a thermoshaker (MHL23, Hettich Benelux). To follow the reaction progress, aliquots of 100 µL were taken after 0, 10, 30, 60, 120 and 180 min. The aliquots were diluted with 1 mL of distilled water and placed immediately in a freezer at −18 °C to quench any ongoing reactions. Sampled aliquots were analyzed via high-performance liquid chromatography (HPLC) with photodiode-array UV-vis detection (Jasco MD-2018 Plus, Tokyo, Japan). A reverse-phase phenyl-hexyl column (100 × 4.6 mm, 2.6 µm particle size) was used, with a 30/70 mixture of acetonitrile/HCl_aq_ (0.05 mmol/L) as an eluent. Chromatograms showed two main peaks. The compounds were identified via comparison with such pure standards as 4-nitrophenol (adsorption maximum at 317 nm, retention time *R*_t_ = 3 min) and 4-aminophenol (absorption maximum at 273 nm, retention time *R*_t_ = 1 min).

## 3. Results and Discussion

### 3.1. Preparation of Polysaccharide/Silica Microcapsules by Spray Drying

Ionically cross-linked polysaccharide microcapsules were obtained via spray-drying in combination with ionic gelation to develop water-resistant capsules. This method is simple, performed under mild conditions, and it can give a reproducible yield. The spontaneous gelation reaction was based on the electrostatic attraction between oppositely charged groups of the macromolecules and the cross-linking agent: either amino groups of chitosan and the polyanion triphosphate, or the carboxylate groups of alginate and calcium ions. A three-dimensional entanglement is precipitated from an aqueous solution in the form of hydrogel microparticles.

The different compositions of the microcapsules prepared in this work are summarized in Table 1. Chitosan samples were cross-linked with sodium triphosphate (STP) and were prepared either without loading (for subsequent post-loading of palladium, samples **F1** and **F4**) or loaded in situ with the hydrophilic compound erioglaucine (samples **F5** to **F7**). Alginate samples, labeled **F8** to **F11**, were cross-linked with calcium chloride (CaCl_2_). Tetraethyl orthosilicate (TEOS) was used as a silica precursor. The hybrid polysaccharide/silica systems were prepared with a polymer-to-TEOS weight ratio of 3:4.

**Figure 1:**
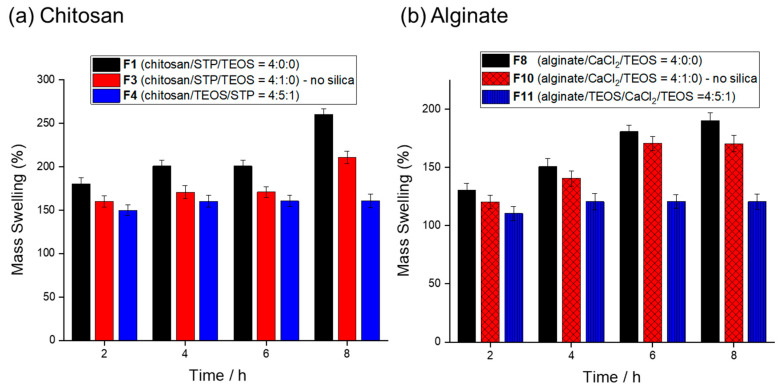
Swelling capacity of (**a**) chitosan and (**b**) alginate microcapsules at different times, as prepared by spray-drying with a polymer–cross-linker–TEOS weight ratio of 4:1:5 in a neutral immersion medium (pH = 7.4).

The swelling capacity of the spray-dried microparticles was studied in buffered phosphate media (pH = 7.4). The corresponding results, plotted as the percentage of mass swelling versus time, are presented in Figure 1. In general, lower water uptake values were observed in the samples containing silica, which retards the dissolution of the polymeric material and increases the structural stability. The swelling capacity of the chitosan microparticles cross-linked with STP decreases considerably when compared with the non-cross-linked ones. The water uptake in hydrogels depends on the extent of the hydrodynamic free volume and the availability of hydrophilic functional groups to establish hydrogen bonds with the water. The swelling behavior and the stability of alginate particles showed the same trend, but the water uptake values were lower even in the absence of silica, which is attributed to the different chemistry of alginate and the stronger interaction between Ca^2+^ ions and the carboxylic groups of alginate.

**Figure 2:**
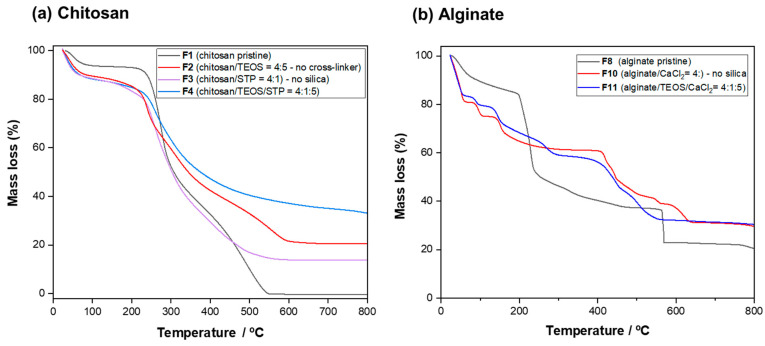
TGA traces of (**a**) chitosan and (**b**) alginate spray-dried microparticles with a polymer–cross-linker–TEOS weight ratio of 4:1:5 (*w*/*w*). Pristine polymers and spray-dried non-cross-linked samples are also shown for comparison (black lines).

Thermogravimetric analysis (TGA) was carried out to evaluate the decomposition of the materials and to determine the silica content. The corresponding thermograms for chitosan and alginate samples are presented in Figure 2. Pristine microparticles, prepared without cross-linker and without silica (**F1** for chitosan and **F8** for alginate), are included for comparison with the hybrid systems. For all samples, mass losses below 100 °C are related to dehydration processes. Under the air atmosphere in which measurements were conducted, the cross-linked chitosan microparticles of sample **F3** (without silica) degrade completely to water and CO_2_, and the final residue corresponds to sodium triphosphate (around 14 wt%). The sample containing silica with no cross-linking agent (sample **F2**) exhibits a silica content around 20 wt%, as estimated from the residue at 800 °C. The residue of sample **F4**, containing silica and the cross-linking agent, is around 34 wt%, consistent with the addition of the residues of samples **F2** and **F3**. Differing from chitosan, alginate is used in the form of a sodium salt, so that there is a Na_2_O residue at high temperatures (about 20% for the pristine sample). The decomposition traces of alginate samples show a complex behavior, with many decomposition steps, in part explained by the presence of hydrated CaCl_2_, which can pass through different hydration states and undergo decomposition at temperatures much lower than the anhydrous form. At 800 °C, the final residues are very similar for the samples without and with silica (samples **F10** and **F11**, respectively), although the TGA traces show considerable differences.

**Figure 3:**
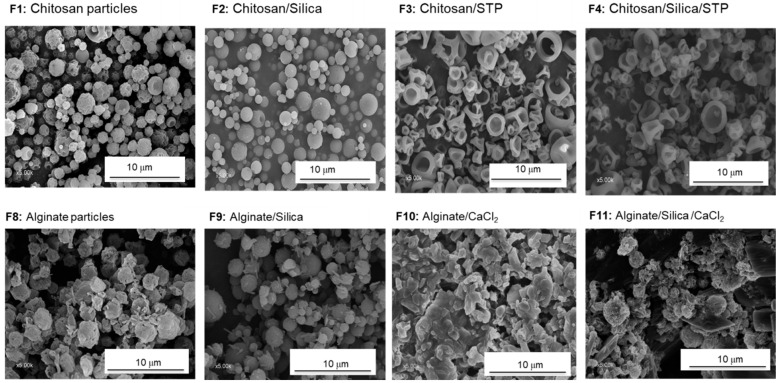
SEM images of spray-dried polysaccharide microparticles in the presence of silica with a polymer–TEOS ratio of 3:4 (samples **F2**–**F4** and **F9**–**F11**). Samples **F1** and **F8** are the control sample prepared without a cross-linker.

The morphology of the different formulations of polysaccharide and polysaccharide/silica hybrid microparticles was studied by means of scanning electron microscopy (SEM). The corresponding micrographs, presented in Figure 3, indicate that the non-cross-linked spray-dried chitosan microspheres (control sample **F1**) are irregular with wrinkles on their surface, most likely as a result of the collapse of the polymer’s hollow structure. Chitosan/silica and dye-loaded chitosan/silica hybrid microparticles (samples **F2** and **F5**) prepared without adding the cross-linker generated well-formed spherical particles with a certain roughness on the surface, as a result of the presence of silica nanostructures embedded within the chitosan matrix. The formulation parameters, namely the dye loading, concentration, and molecular weight of chitosan, induced a remarkable change in the surface morphology of the microspheres. The cross-linked particles (samples **F3, F4** and **F6**) show different morphologies when compared to the non-cross-linked ones. The addition of STP favored the formation of a shrunken morphology with partial roughness as a result of the higher amount of water content (moisture) inside the particles. This morphology is attributed to the rapid evaporation during the first stages of the drying process.

Alginate non-cross-linked microparticles (control sample **F8**) also show an irregular, non-spherical structure. Similar to chitosan systems, without addition of the cross-linker, the presence of silica (samples **F9**) resulted in a more spherical morphology. When calcium chloride was added as a cross-linker (samples **F10** and **F11**), the morphology became more irregular, and calcium chloride crystals are sporadically observed, even after several washing steps. Overall, when compared to chitosan, under similar operation conditions, alginate microparticles showed a less homogeneous and controllable morphology. Therefore, only chitosan systems were selected in the rest of the work for the application experiments, both for release of hydrophilic substances and catalytic experiments. Nevertheless, a further optimization of the conditions for the alginate system could also potentially lead to comparable results.

### 3.2. Application of Chitosan Microcapsules as Carriers of Hydrophilic Substances

The spray-dried chitosan microparticles were loaded with a model hydrophilic substance (erioglaucine disodium salt). A definite amount (2 wt%) with respect to the polymer weight was mixed with the chitosan solution, with or without the presence of TEOS. After dissolution of the dye, the cross-linker solution was added to these mixtures, followed by spray-drying, yielding the dye-loaded microparticles.

**Figure 4:**
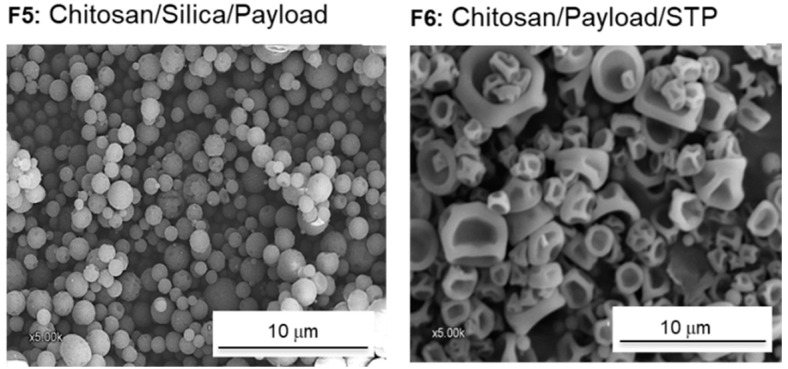
SEM images of spray-dried polysaccharide microparticles loaded with erioglaucine in the presence of silica with a polymer–TEOS ratio 3:4 (samples **F5** and **F6**).

The hydrophilic molecules were encapsulated with different formulations in the presence (samples **F5** and **F7**) and in the absence of silica (sample **F6**) to estimate the effect of silica nanostructures on the release behavior, in a comparison study with the non-cross-linked ones. The morphology of the obtained microparticles was similar to those obtained in the absence of erioglaucine, as seen in the SEM micrographs of Figure 4.

**Figure 5:**
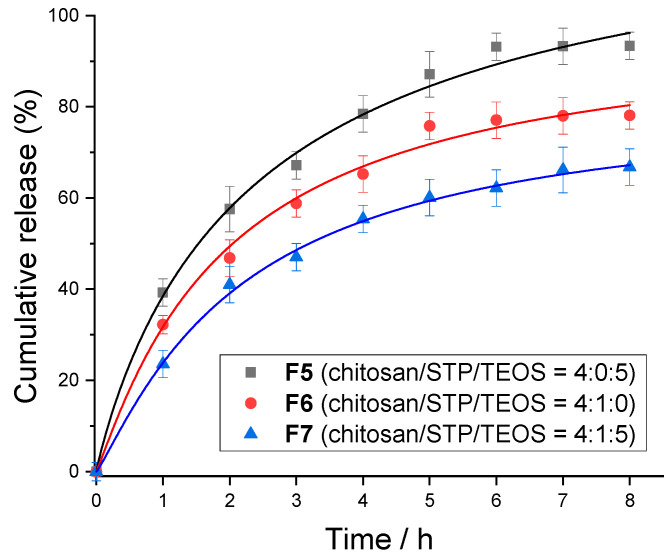
Cumulative release of erioglaucine loaded in sample **F5** (chitosan/silica hybrid spray-dried microparticles without cross-linker), sample **F6** (chitosan cross-linked spray-dried particles without silica), and sample **F7** (chitosan/silica hybrid cross-linked microparticles in phosphate buffer, pH = 7.4). The solid lines represent the theoretical fitting according to the Hill equation.

Release behavior was studied by performing dissolution studies in a phosphate-buffered solution (pH = 7.4) at 37 °C. Figure 5 presents the release process of spray-dried chitosan–STP microparticles. The overall release takes place rapidly during the first 3 h (approximately 40% to 60%), which can be attributed to the presence of a sufficient amount of the erioglaucine, which adheres to the microcapsules’ surface during the drying steps. Overall, the release is governed by the diffusion ability as a result of the swelling process. The data are fitted to the Hill equation, analogously to our previous work [27].

The release of the dye from the non-cross-linked microparticles was higher in the presence of silica (sample **F5**). It is clearly slower in the absence of silica for the cross-linked particles (sample **F6**). This behavior is mainly influenced by the cross-linking density of the cross-linker (STP) in chitosan, which decreases the swelling capacity of the particles due to the presence of a stronger matrix. On the other hand, chitosan/silica hybrid cross-linked microparticles (sample **F7**) are more stable, and the release is much slower in comparison with the non-cross-linked ones under the same conditions. This observation is explained by both the strong complexation of the dye to the chitosan and the adsorption on silica. The results reveal that the hybrid chitosan/silica cross-linked microparticles are more efficient than the non-cross-linked ones in encapsulating erioglaucine, as well as retarding its release.

### 3.3. Application of Chitosan Microcapsules in Catalysis

In a second part of the work, we studied the use the prepared materials for heterogeneous catalysis. Specifically, we deposited Pd(0) nanoparticles on spray-dried microparticles. The resulting palladium-containing samples were used as catalysts for the reduction of 4-nitrophenol with NaBH4. We applied a “post-loading” approach, in which the Pd(0) deposition took place after the formation of the chitosan particles. Two samples were loaded, one without silica (**F3**) and one with silica (**F4**). In our process, the particles were suspended in a hydrochloric acid solution of PdCl_2_, the pH value of the acidic PdCl_2_ suspension was increased from 1 to 12, and the particles were separated after stirring for a certain time. Pd(II) was then reduced to Pd(0) with NaBH_4_.

**Figure 6:**
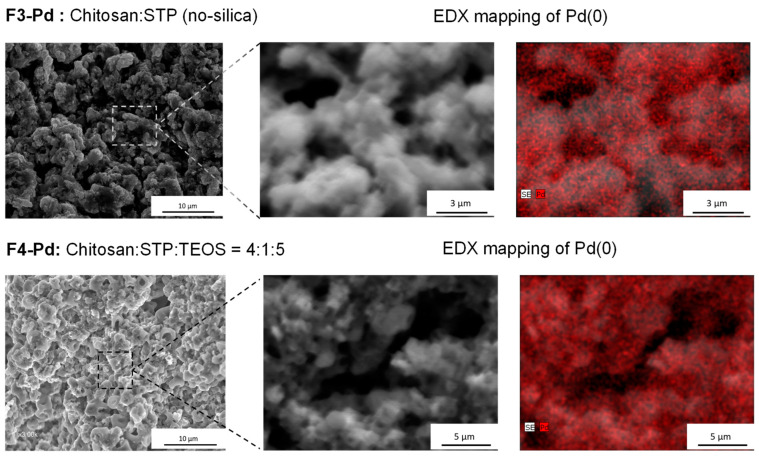
SEM images and EDX elemental mapping of the catalysts **F3-Pd** (without silica) and **F4-Pd** (with silica).

Figure 6 shows SEM images of catalysts **F3-Pd** (without silica) and **F4-Pd** (with silica). The micrographs show that particles without silica tend to aggregate after the post-loading process, while individual particles are distinguishable with silica (sample **F4-Pd**). The relative molar concentration of Pd normalized to the carbon value was determined via energy dispersive X-ray spectroscopy (EDX) during the SEM measurements. The measured Pd quantities were 19.3 mol% and 11.6 mol% for the samples without silica and with silica, respectively. The surface area of the Pd-loaded microparticles, as determined from BET isotherms for nitrogen adsorption–desorption, was less under 1 m^2^/g for all samples. In spite of this low surface area, in principle expectable for particles of micrometer size, significant catalytic activity was found in the hybrid palladium/chitosan systems, as shown below.

**Scheme 1:**
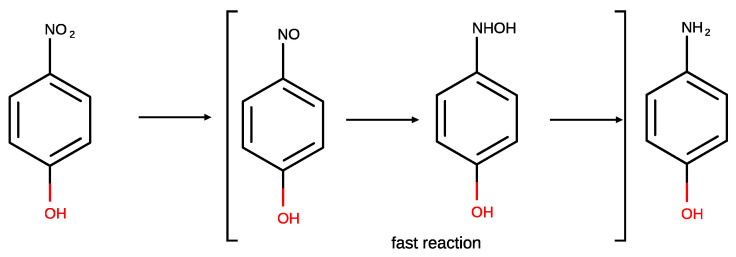
Direct Haber pathway for 4-nitrophenol.

Formally, the hydrogenation of 4-nitrophenol in diluted solution can be described using the simplified process shown in Scheme 1, based on the model of Haber [40,41]. This pathway consists of three consecutive reactions, leading to the formation of 4-aminophenol from *N*-(4-hidroxyphenyl)hydroxylamine through the corresponding nitroso derivative. The literature suggests that reduction of 4-nitrophenol with borohydride is simple enough for use as a suitable model for activity comparison [42,43,44,45]. For this system, the intermediate products (i.e., *N*-(4-hydroxyphenyl)hydroxylamine and 4-nitrosophenol) reduce so rapidly that only 4-aminophenol is detected. Despite its apparent simplicity, the rate law for this heterogeneous reduction depends heavily on the catalyst used. In this regard, the reaction is usually described as first-order, but there is strong evidence that the rate law is more complicated due to the nitroarene and hydride adsorption phenomena on the Pd surface [46,47,48].

**Figure 7:**
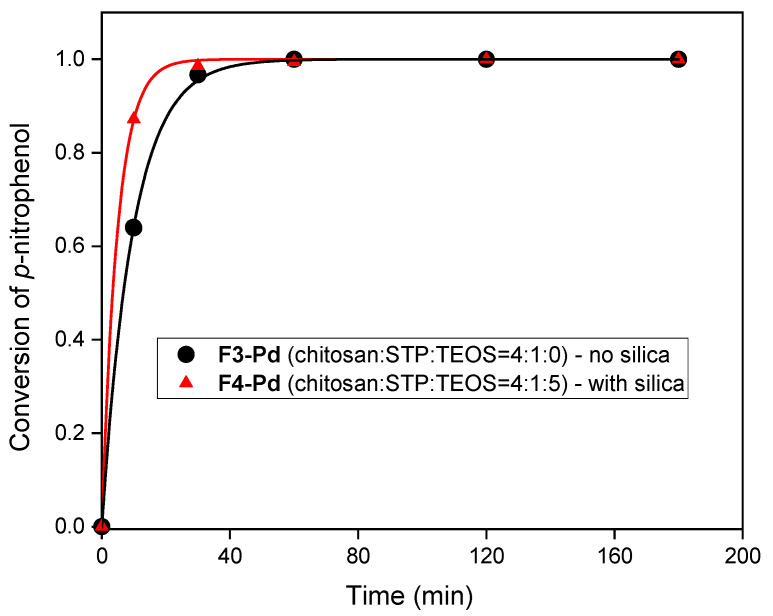
Conversion of 4-nitrophenol during a given reaction time, together with least-squares exponential plots according to Equation (4).

The conversion of 4-nitrophenol throughout the reaction time is shown in Figure 7, and calculated according to Equation (3):

(3) $$x=1-\frac{{\left[NP\right]}_{t}}{{\left[NP\right]}_{0}}=1-\frac{A_{t}-A_{\infty }}{A_{0}-A_{\infty }}$$

where [NP]*_t_* and [NP]_0_ are the 4-nitrophenol concentrations at times *t* and zero, respectively; *A_t_* stands for the absorbance at 317 nm at time *t*, and *A*_0_ and *A*_∞_ are the absorbances at zero time and by the end of reaction, respectively. Assuming a first-order kinetics, the change of *x* with time is given by Equation (4),

(4) $$x\left(t\right)=1-e^{-kt}$$

where *k* is the pseudo-first order rate constant for the direct conversion to 4-aminophenol. It should be noted that pseudo-first order conditions are achieved, as reactions are conducted in a large NaBH_4_ excess. Consequently, this coefficient depends on the reducing agent concentration. The *k* values were calculated by the non-linear least-squares fitting of experimental *x* data to those calculated from Equation (5). The results of the data analysis are collected in Table 2. The reaction catalyzed by microparticles exhibited significantly high *k* values, in agreement with a very fast kinetic with very high conversion within quite short times, as shown in the conversion graphs.

To compare the activity of palladium deposited on different carriers using different post-loading methods, the turn-over frequency (TOF) was calculated. TOF is defined as the quotient between the substrate disappearance rate and the active center concentration, in this case the quantity of Pd placed in the reactor (Equation (5)):

(5) $$TOF=-\frac{r_{NP}}{\left[Pd\right]}$$

Since the TOF value is time-dependent through *r*_NP_, we will take the half-reaction time (*t*_1/2_) as a reference. On the other hand, to avoid the use of a particular kinetic model, we will use the “corrected” TOF time average calculated at half-reaction time (Equation (6)), corrected by the co-reactant concentration excess (i.e., the initial concentration of sodium borohydride, [NaBH_4_]_0_):

(6) $${TOF}_{\frac{1}{2}\;(corr)}=\frac{0.5{\left[NP\right]}_{0}}{t_{\frac{1}{2}}\left[\mathrm{Pd}\right]{\left[NaBH_{4}\right]}_{0}}$$

The experimental half-life time *t*_1/2_ was determined using linear interpolation. The TOF_1/2 (corr)_ values are 0.03 and 0.06 for samples **F3-Pd** (without silica) and **F4-Pd** (with silica), respectively. The obtained values are within the common ranges for heterogenized palladium nanoparticles. A previous work compared the values of *k*, *t*_1/2_, and TOF_1/2_ for _¡_a wide variety of supported Pd nanoparticles from the literature (cf. Table 4 in ref. [48]). In general, the comparison of results from different works is not trivial, as the conditions are not necessarily comparable. However, the results achieved in this work indicate that our method is able to yield very efficient heterogeneous catalysts, produced in a simple and reproducible way, while using an economic and biocompatible support (chitosan or chitosan/silica). When compared with the chitosan-only particles, the inclusion of silica has higher structural stability as an advantage, because the catalysts can be more easily separated from the medium, with less degradation.

## 4. Conclusions

The present work demonstrates the feasibility of the spray-drying method for encapsulating hydrophilic molecules (erioglaucine sodium salt) into chitosan–STP microparticles prepared through ionotropic gelation. This method was selected because it is reproducible, rapid, and relatively easy to scale up. The applied preparation process favored the formation of shrunken microparticles with a rough surface. The results indicated that the cross-linked hybrid chitosan/silica microparticles prepared using this process retarded the release behavior in the neutral medium in comparison with the non-cross-linked microparticles. They also showed very good catalytic performance for the reduction of *p*-nitrophenol by NaBH_4_. In addition to their easy preparation, the microparticles are made of biocompatible and readily available compounds (chitosan or alginate, and silica), which may be a significant advantage when compared to other materials used for the same applications.

## Figures and Tables

**Table 1:** Compositions of the prepared polysaccharide and polysaccharide/silica microparticles.

Sample	System	Cross-Linker	Polymer:Cross-Linker:TEOS Weight Ratio	Initial Load (In Situ) ^[a]^
**F1**	Chitosan	—	4:0:0	—
**F2**	Chitosan/silica	—	4:0:5	—
**F3**	Chitosan	STP	4:1:0	—
**F4**	Chitosan/silica	STP	4:1:5	—
**F5**	Chitosan/silica	—	4:0:5	Erioglaucine
**F6**	Chitosan	STP	4:1:0	Erioglaucine
**F7**	Chitosan/silica	STP	4:1:5	Erioglaucine
**F8**	Alginate	—	4:0:0	—
**F9**	Alginate/silica	—	4:0:5	—
**F10**	Alginate	CaCl_2_	4:1:0	—
**F11**	Alginate/silica	CaCl_2_	4:1:5	—

^[a]^ Samples **F3** and **F4** of chitosan were subsequently post-loaded with palladium for use in catalytic applications.

**Table 2:** Activity parameters for catalysts **F3-Pd** (without silica) and **F4-Pd** (with silica).

Catalyst	${TOF}_{\frac{1}{2}(corr)}$/min^−1^ mol^−1^ L	*k*/min^−1^	t_1/2_/min
**F3-Pd**	0.03	0.10	6.7
**F4-Pd**	0.06	0.21	3.4

## Data Availability

The data presented in this study are available upon request from the corresponding author.

## References

[1] Kumar S., Shen J., Zolnik B., Sadrieh N., Burgess D.J. (2015). Optimization and dissolution performance of spray-dried naproxen nano-crystals. Int. J. Pharm..

[2] Lee S.H., Heng D., Ng W.K., Chan H.-K., Tan R.B.H. (2011). Nano spray drying: A novel method for preparing protein nanoparticles for protein therapy. Int. J. Pharm..

[3] Abdel Bary E., Fekri A., Soliman Y., Harmal A. (2016). Biodegradable polymer nanocomposites based on polyvinyl alcohol and nano-rice straw. Indian J. Appl. Res..

[4] Ammala A. (2013). Biodegradable polymers as encapsulation materials for cosmetics and personal care markets. Int. J. Cosm. Sci..

[5] Aranaz I., Paños I., Peniche C., Heras Á., Acosta N. (2017). Chitosan spray-dried microparticles for controlled delivery of venlafaxine hydrochloride. Molecules.

[6] Schmid K., Arpagaus C., Friess W. (2011). Evaluation of the nano spray dryer b-90 for pharmaceutical applications. Pharm. Dev. Technol..

[7] Li X., Anton N., Arpagaus C., Belleteix F., Vandamme T.F. (2010). Nanoparticles by spray drying using innovative new technology: The büchi nano spray dryer b-90. J. Control. Release.

[8] Kašpar O., Jakubec M., Štěpánek F. (2013). Characterization of spray dried chitosan–tpp microparticles formed by two- and three-fluid nozzles. Powder Technol..

[9] Liu W., Chen X.D., Selomulya C. (2015). On the spray drying of uniform functional microparticles. Particuology.

[10] Wan F., Yang M. (2016). Design of plga-based depot delivery systems for biopharmaceuticals prepared by spray drying. Int. J. Pharm..

[11] Kulkarni A.D., Bari D.B., Surana S.J., Pardeshi C.V. (2016). In vitro, ex vivo and in vivo performance of chitosan-based spray-dried nasal mucoadhesive microspheres of diltiazem hydrochloride. J. Drug Deliv. Sci. Technol..

[12] Kumar S., Shen J., Burgess D.J. (2014). Nano-amorphous spray dried powder to improve oral bioavailability of itraconazole. J. Control. Release.

[13] Klein M.P., Hackenhaar C.R., Lorenzoni A.S.G., Rodrigues R.C., Costa T.M.H., Ninow J.L., Hertz P.F. (2016). Chitosan crosslinked with genipin as support matrix for application in food process: Support characterization and β-d-galactosidase immobilization. Carbohydr. Polym..

[14] Sung H.-W., Huang R.-N., Huang L.L.H., Tsai C.-C. (1999). In vitro evaluation of cytotoxicity of a naturally occurring cross-linking reagent for biological tissue fixation. J. Biomater. Sci. Polym. Ed..

[15] Panos I., Acosta N., Heras A. (2008). New drug delivery systems based on chitosan. Curr. Drug Discov. Technol..

[16] Sreekumar S., Lemke P., Moerschbacher B.M., Torres-Giner S., Lagaron J.M. (2017). Preparation and optimization of submicron chitosan capsules by water-based electrospraying for food and bioactive packaging applications. Food Addit. Contam. Part A Chem. Anal..

[17] Anal A.K., Stevens W.F., Remunan-Lopez C. (2006). Ionotropic cross-linked chitosan microspheres for controlled release of ampicillin. Int. J. Pharm..

[18] Blandino A., Macías M., Cantero D. (1999). Formation of calcium alginate gel capsules: Influence of sodium alginate and cacl2 concentration on gelation kinetics. J. Biosci. Bioeng..

[19] Wang S., Chen M., Wu L. (2016). One-step synthesis of cagelike hollow silica spheres with large through-holes for macromolecule delivery. ACS Appl. Mater. Interfaces.

[20] Doan-Nguyen T.P., Natsathaporn P., Jenjob R., Niyom Y., Ittisanronnachai S., Flood A., Crespy D. (2019). Regulating payload release from hybrid nanocapsules with dual silica/polycaprolactone shells. Langmuir.

[21] Fan J., Wang S., Sun W., Guo S., Kang Y., Du J., Peng X. (2018). Anticancer drug delivery systems based on inorganic nanocarriers with fluorescent tracers. AlChE J..

[22] Ilhan-Ayisigi E., Yesil-Celiktas O. (2018). Silica-based organic-inorganic hybrid nanoparticles and nanoconjugates for improved anticancer drug delivery. Eng. Life Sci..

[23] Niyom Y., Phakkeeree T., Flood A., Crespy D. (2019). Synergy between polymer crystallinity and nanoparticles size for payloads release. J. Colloid Interface Sci..

[24] Gaitzsch J., Huang X., Voit B. (2016). Engineering functional polymer capsules toward smart nanoreactors. Chem. Rev..

[25] Behzadi S., Steinmann M., Estupiñán D., Landfester K., Crespy D. (2016). The pro-active payload strategy significantly increases selective release from mesoporous nanocapsules. J. Control. Release.

[26] Hajir M., Dolcet P., Fischer V., Holzinger J., Landfester K., Muñoz-Espí R. (2012). Sol–gel processes at the droplet interface: Hydrous zirconia and hafnia nanocapsules by interfacial inorganic polycondensation. J. Mater. Chem..

[27] Elzayat A., Tolba E., Pérez-Pla F.F., Oraby A., Muñoz-Espí R. (2021). Increased stability of polysaccharide/silica hybrid sub-millicarriers for retarded release of hydrophilic substances. Macromol. Chem. Phys..

[28] Shen C., Wang Y.J., Xu J.H., Wang K., Luo G.S. (2012). Size control and catalytic activity of highly dispersed pd nanoparticles supported on porous glass beads. Langmuir.

[29] Montsch T., Heuchel M., Traa Y., Klemm E., Stubenrauch C. (2017). Selective hydrogenation of 3-hexyn-1-ol with pd nanoparticles synthesized via microemulsions. Appl. Catal. A.

[30] Thompson S.T., Lamb H.H. (2017). Catalysts for selective hydrogenation of furfural derived from the double complex salt [pd(nh3)4](reo4)2 on γ-al2o3. J. Catal..

[31] Djoković V., Božanic D.K., Vodnik V.V., Krsmanović R.M., Trandafilovic L.V., Dimitrijević-Branković S. (2011). Structure and Optical Properties of Noble-Metal and Oxide Nanoparticles Dispersed in Various Polysaccharide Biopolymers.

[32] Liao X., Zhang Y., Hill M., Xia X., Zhao Y., Jiang Z. (2014). Highly efficient ni/ceo2 catalyst for the liquid phase hydrogenation of maleic anhydride. Appl. Catal. A.

[33] Regenhardt S.A., Meyer C.I., Garetto T.F., Marchi A.J. (2012). Selective gas phase hydrogenation of maleic anhydride over ni-supported catalysts: Effect of support on the catalytic performance. Appl. Catal. A.

[34] Liu G., Hou M., Song J., Jiang T., Fan H., Zhang Z., Han B. (2010). Immobilization of pd nanoparticles with functional ionic liquid grafted onto cross-linked polymer for solvent-free heck reaction. Green Chem..

[35] Berguerand C., Yuranov I., Cárdenas-Lizana F., Yuranova T., Kiwi-Minsker L. (2014). Size-controlled pd nanoparticles in 2-butyne-1,4-diol hydrogenation: Support effect and kinetics study. J. Phys. Chem. C.

[36] Leonhardt S.E.S., Stolle A., Ondruschka B., Cravotto G., Leo C.D., Jandt K.D., Keller T.F. (2010). Chitosan as a support for heterogeneous pd catalysts in liquid phase catalysis. Appl. Catal. A.

[37] Elzayat A.M., Pérez-Pla F.F., Muñoz-Espí R. (2022). A chitosan/silica hybrid 3d scaffold for simultaneous entrapment of two different hydrophilic substances. Mater. Lett..

[38] Goutelle S., Maurin M., Rougier F., Barbaut X., Bourguignon L., Ducher M., Maire P. (2008). The hill equation: A review of its capabilities in pharmacological modelling. Fundam. Clin. Pharmacol..

[39] Murzin D.Y., Heikkilä T. (2014). Modeling of drug dissolution kinetics with sigmoidal behavior from ordered mesoporous silica. Chem. Eng. Commun..

[40] Haber F. (1898). Gradual electrolytic reduction of nitrobenzene with limited cathode potential. Elektrochem. Angew. Phys. Chem.

[41] Corma A., Concepción P., Serna P. (2007). A different reaction pathway for the reduction of aromatic nitro compounds on gold catalysts. Ang. Chem. Int. Ed..

[42] Ye W., Yu J., Zhou Y., Gao D., Wang D., Wang C., Xue D. (2016). Green synthesis of pt–au dendrimer-like nanoparticles supported on polydopamine-functionalized graphene and their high performance toward 4- nitrophenol reduction. Appl. Catal. B.

[43] Movahed S.K., Lehi N.F., Dabiri M. (2018). Palladium nanoparticles supported on core-shell and yolk-shell fe3o4@nitrogen doped carbon cubes as a highly efficient, magnetically separable catalyst for the reduction of nitroarenes and the oxidation of alcohols. J. Catal..

[44] Zhao P., Feng X., Huang D., Yang G., Astruc D. (2015). Basic concepts and recent advances in nitrophenol reduction by gold- and other transition metal nanoparticles. Coord. Chem. Rev..

[45] Gu S., Wunder S., Lu Y., Ballauff M., Fenger R., Rademann K., Jaquet B., Zaccone A. (2014). Kinetic analysis of the catalytic reduction of 4-nitrophenol by metallic nanoparticles. J. Phys. Chem. C.

[46] Lara L.R.S., Zottis A.D., Elias W.C., Faggion D., Maduro de Campos C.E., Acuña J.J.S., Domingos J.B. (2015). The catalytic evaluation of in situ grown pd nanoparticles on the surface of fe3o4@dextran particles in the p-nitrophenol reduction reaction. RSC Adv..

[47] Aditya T., Pal A., Pal T. (2015). Nitroarene reduction: A trusted model reaction to test nanoparticle catalysts. Chem. Commun..

[48] Ródenas M., El Haskouri J., Ros-Lis J.V., Marcos M.D., Amorós P., Úbeda M.Á., Pérez-Pla F. (2020). Highly active hydrogenation catalysts based on pd nanoparticles dispersed along hierarchical porous silica covered with polydopamine as interfacial glue. Catalysts.

